# The combined effect of subcutaneous granulocyte- colony stimulating factor and myocardial contrast echocardiography with intravenous infusion of sulfur hexafluoride on post-infarction left ventricular function, the RIGENERA 2.0 trial: study protocol for a randomized controlled trial

**DOI:** 10.1186/s13063-016-1172-0

**Published:** 2016-02-19

**Authors:** Antonio Maria Leone, Domenico D’Amario, Luciana Teofili, Eloisa Basile, Francesco Cannata, Francesca Graziani, Mario Marzilli, Antonio Matteo Russo, Giuseppe Tarantini, Claudio Ceconi, Giuseppe Leone, Carlo Trani, Antonio Giuseppe Rebuzzi, Filippo Crea

**Affiliations:** Dipartimento di Scienze Cardiovascolari, Università Cattolica del Sacro Cuore, Largo Agostino Gemelli, 8, Rome, 00168 Italy; Istituto di Ematologia, Università Cattolica del Sacro Cuore, Largo Agostino Gemelli, 8, Rome, 00168 Italy; Dipartimento Cardio-Toraco-Vascolare, Università di Pisa, Via Paradisa, 2, Pisa, 56124 Italy; MEBIC, Università San Raffaele, Via di Val Cannuta, 247 - 00166, Rome, Italy; Dipartimento di Scienze Cardiologiche Toraciche e Vascolari, Università di Padova, Via Giustiniani, 2, Padova, 35128 Italy; Dipartimento di Medicina Clinica e Sperimentale, Università di Ferrara, Corso Giovecca, 203, Ferrara, 44100 Italy

**Keywords:** ST-elevation myocardial infarction (STEMI), Granulocyte-colony stimulating factor (G-CSF), Bone marrow-derived stem cells, Myocardial contrast echocardiography (MCE), Remodeling, Post-myocardial infarction heart failure

## Abstract

**Background:**

Several clinical trials and recent meta-analyses have demonstrated that administration of recombinant human granulocyte-colony stimulating factor (G-CSF) is safe and, only in patients with large acute myocardial infarction (AMI), is associated with an improvement in left ventricular ejection fraction. Moreover, the mobilization and engraftment of the bone marrow-derived cells may differ significantly among patients, interfering with the restoration of left ventricular function after treatment. Therefore, the clinical potential application of the G-CSF has not yet been fully elucidated.

**Methods/Design:**

The RIGENERA 2.0 trial is a multicenter, phase II, placebo-controlled, randomized, open-label, with blinded evaluation of endpoints (PROBE) trial in which 120 patients with an acute ST-elevation myocardial infarction (STEMI) undergoing successful revascularization but with residual myocardial dysfunction will be enrolled. In cases where there is a left ventricular ejection fraction (LVEF) ≤45 % the patient will be electronically randomized (1:1 ratio) to receive either subcutaneous recombinant human G-CSF (group 1) or placebo (group 2) both added on top of optimal standard of care. Both groups will undergo myocardial contrast echocardiography with intravenous infusion of sulfur hexafluoride (MCE) whilst undergoing the echocardiogram.

The primary efficacy endpoint is the evaluation of the LVEF at 6 months after AMI assessed by cardiac magnetic resonance. Secondary efficacy endpoints are the evaluation of LVEF at 6 months after AMI assessed by echocardiography, left ventricular end-diastolic volume (LVEDV) and left ventricular end-systolic volume (LVESV) assessed by cardiac magnetic resonance and echocardiography at 6 months, together with the incidence of major adverse clinical events (MACE) defined as death, myocardial infarction, sustained cardiac arrhythmias, cardiogenic shock, stroke and re-hospitalization due to heart failure at 1 year.

**Discussion:**

The RIGENERA 2.0 trial will test whether G-CSF administration and MCE, through the enhancement of the bone marrow-derived cells homing in the myocardium, determines an improvement in regional and global contractile function, myocardial perfusion and infarct extension in patients with large AMI. The results of the present study are expected to envision routine clinical use of this safe, affordable and reproducible approach in patients with successful revascularization after AMI.

**Trial registration:**

ClinicalTrials.gov: NCT02502747 (29 June 2015); EudraCT: 2015-002189-21 (10 July 2015).

**Electronic supplementary material:**

The online version of this article (doi:10.1186/s13063-016-1172-0) contains supplementary material, which is available to authorized users.

## Background

The long-term prognosis of patients suffering from acute myocardial infarction (AMI) has progressively improved since the introduction of reperfusion therapies and, in particular, of primary angioplasty [1]. In the setting of ST-elevation myocardial infarction (STEMI), the immediate reopening of acutely occluded coronary arteries via primary angioplasty is the treatment of choice to salvage the ischemic myocardium. However, the sudden re-initiation of blood flow can lead to a local acute inflammatory response with further endothelial and myocardial damage. This phenomenon, described as “reperfusion injury”, may explain why, despite optimum myocardial reperfusion, the short-term mortality after AMI approaches 7 % [1] and the long-term incidence of heart failure in reported to be between 15 and 20 % [2, 3]. Despite the use of full conventional treatments, including ACE inhibitors, beta-blockers, aldosterone inhibitors and diuretics, in the context of randomized controlled trials, yearly mortality rates of patients with post-infarction heart failure are still 10 % and re-hospitalization for worsening of heart failure occurs at a yearly rate of 6–8 % [4].

A major reason for the high morbidity and mortality is that the heart has an inadequate regenerative response to the myocardial necrosis following AMI; cell death from the ischemic damage can lead to progressive ventricular dilation and dysfunction (i.e., adverse left ventricular remodeling). Numerous pre-clinical studies and small to intermediate size clinical trials have demonstrated beneficial effects of bone marrow-derived cells on top of state-of-the-art reperfusion treatment providing compelling evidence that bone marrow- derived cells do contribute to cardiac repair after acute myocardial injury, limiting infarct expansion and improving cardiac function, most likely via paracrine mechanisms [5–7].

Considering that recovery of post-infarction left ventricular function is favorably correlated with bone marrow-derived stem cell mobilization induced by endogenous granulocyte-colony stimulating factor (G-CSF) [8, 9], therapeutic stem cell mobilization has been proposed as a therapeutic tool. Several basic studies have supported this notion suggesting that stem cell mobilization might improve left ventricular function by increasing stem cell homing in the heart in addition to a favorable direct effect of G-CSF on the injured myocardium [10–12].

However, the clinical effects of G-CSF on patients with AMI have so far shown mixed results [13]: G-CSF was proven to be safe but the therapeutic effect appeared modest. Nevertheless, in patients with large myocardial infarction the effect was clinically relevant, approaching a 5 % increase in left ventricular ejection fraction (LVEF). In particular, in the RIGENERA trial conducted by our group, 41 patients with large anterior wall AMI at high risk of unfavorable remodeling were randomized 1:2 to Lenograstim (recombinant human (rhu) G-CSF, Myelostim 34, Italfarmaco, Milan, Italy) (10 μg/kg/day for 5 days) or to conventional therapy. After a median follow-up of 5 months the patients treated with Lenograstim exhibited improvement of 5 % in LVEF, in the absence of left ventricular dilation. In contrast, the patients treated conventionally exhibited significant dilation in the absence of an improvement in LVEF [14]. Moreover, a subgroup of eight patients randomized to Lenograstim were administered a second-generation ultrasound contrast agent containing sulfur hexafluoride (SonoVue®, Bracco, Milan, Italy) administered intravenously (5 ml at 1 ml/min) to achieve myocardial opacification in order to collect diagnostic images by myocardial contrast echocardiography. Interestingly, a post hoc analysis, revealed that patients receiving SonoVue® showed a significant benefit in left ventricular function (unpublished data): the myocardial destruction of contrast medium-microbubbles by 3.5-MHz ultrasound produced by the echo machine may have increased the homing of the mobilized bone marrow-derived stem cells. Additionally, two studies on animal models have consistently demonstrated that the highly focused ultrasound-mediated stimulation of microbubbles increased the migration of stem cells across the myocardial endothelium into the post-ischemic myocardium in vivo [15, 16].

In our study population, the long-term (10 years) follow-up data, recently collected, confirmed the safety of the protocol and, remarkably, patients treated with G-CSF showed a significant improvement of the quality of life assessed by New York Heart Association (NYHA) functional class, Seattle Heart Failure Model, Minnesota Living with Heart Failure Questionnaire (*p* < 0.005) (unpublished data).

Therefore, on the basis of the previous results collected by our group, the RIGENERA 2.0 trial (ClinicalTrials.gov Identifier: NCT02502747; EudraCT number: 2015-002189-21) was designed to specifically test the efficacy of the combined effect of subcutaneous G-CSF and sulfur hexafluoride on post-infarction left ventricular function in patients with STEMI.

## Methods

### Study objectives

The overall objectives of the RIGENERA 2.0 trial are to determine whether, in patients with large AMI undergoing primary or rescue angioplasty, the administration of subcutaneous Lenograstim (rhu G-CSF, Myelostim 34, Italfarmaco, Milan, Italy) associated with myocardial contrast echocardiography and the intravenous infusion of sulfur hexafluoride (SonoVue®, Bracco, Milan, Italy) determines an improvement in regional and global contractile function, myocardial perfusion and infarct size and is associated to a better quality of life.

Specifically, the primary efficacy endpoint is:

- LVEF at 6 months assessed by cardiac magnetic resonance and centrally reviewed.

Secondary efficacy endpoints are:

- Left ventricular end-diastolic volume (LVEDV) assessed by cardiac magnetic resonance at 6 months and centrally reviewed
- Left ventricular end-systolic volume (LVESV) assessed by cardiac magnetic resonance at 6 months and centrally reviewed
- Left ventricular ejection fraction (LVEF) assessed by 2D echocardiography at 6 months and centrally reviewed
- Left ventricular end-diastolic volume (LVEDV) assessed by 2D echocardiography at 6 months and centrally reviewed
- Left ventricular end-systolic volume (LVESV) assessed by 2D echocardiography at 6 months and centrally reviewed
- Incidence of major adverse clinical events (death, myocardial infarction, sustained cardiac arrhythmias, cardiogenic shock, stroke and re-hospitalization due to heart failure at 1 year) (see Additional file 1)

Safety endpoints are*:*

- Incidence of adverse events
- Incidence of new neoplastic and hematological diseases.

### Study design (Fig. 1)

The RIGENERA 2.0 trial is a phase II placebo-controlled, randomized, open-label, with blinded evaluation of endpoints (PROBE) trial. One hundred and twenty STEMI patients are planned to be enrolled in four Italian centers (Catholic University of the Sacred Heart in Rome, University of Ferrara, Pisa and Padua). Patients with an acute STEMI, as defined by the universal definition of AMI, undergoing acute revascularization (i.e., either acute percutaneous coronary intervention (PCI) within 24 hours of symptom onset or thrombolysis within 12 hours followed by acute PCI within 24 hours of symptom onset) will be screened at investigational sites. Patients who have had acute PCI in institutions different from the investigational sites (recruiting centers) can also be included: interested patients may be referred for screening to any of the participating study sites within 3 to 4 days. Every patient will be informed in person and receive the patient information letter and informed consent form; as this trial has not yet begun the recruitment phase, no informed consent form has been provided to any patient; however, informed consent will be obtained from all participants.

**Fig. 1:**
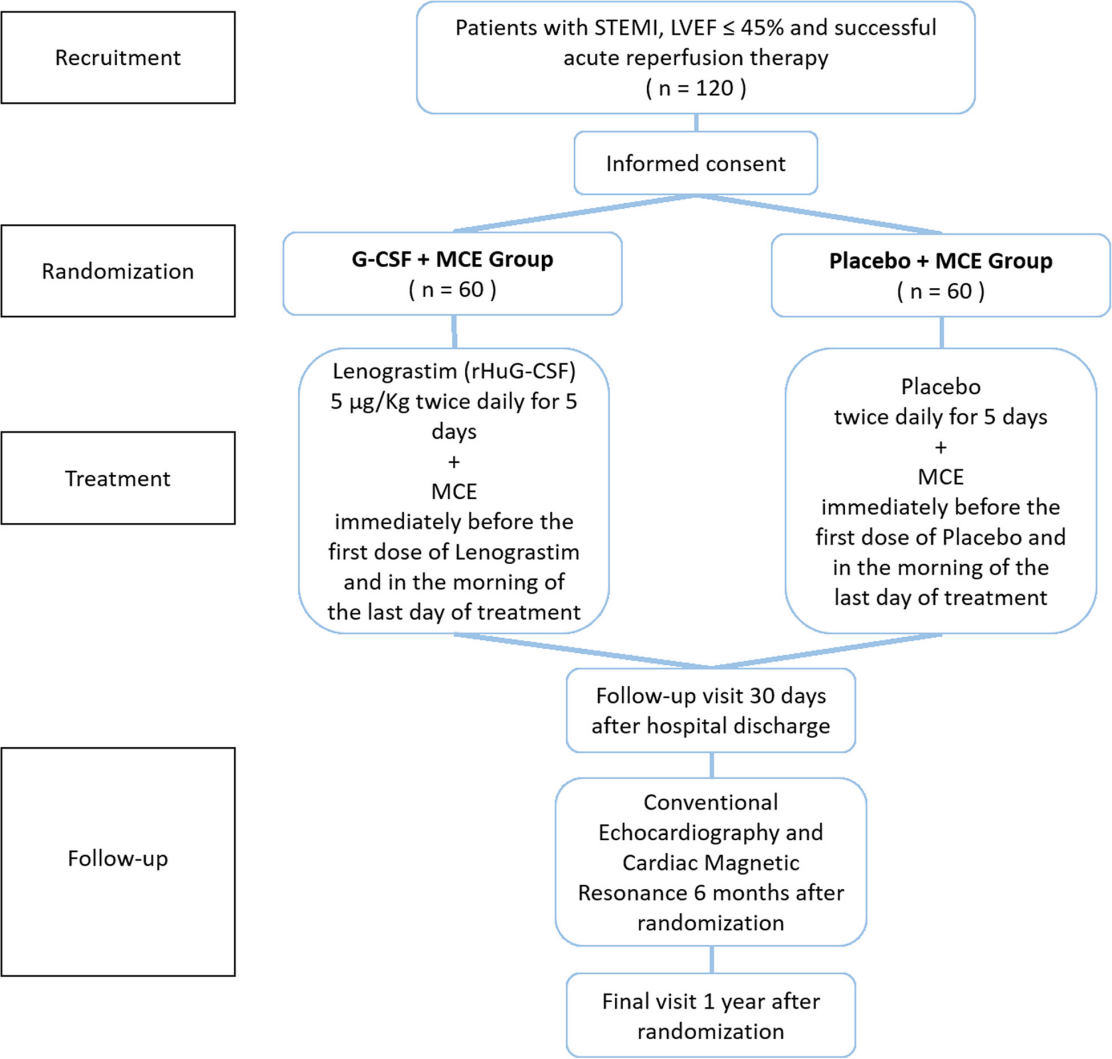
Flow chart of the RIGENERA 2.0 trial. *MCE* myocardial contrast echocardiography

The study was approved by the ethical committee (Comitato Etico del Policlinico Gemelli, Roma) and AIFA (Italian Medicines Agency) in July 2015. The trial will be conducted in accordance with the Italian Medical Drug Law (received the AIFA approval in July 2015) and the Good Clinical Practice guidelines. The RIGENERA 2.0 trial is an investigator-initiated trial and funded by the Italian Ministry of Education and Research (PRIN 2010/2011/2010S7CET4).

Informed consent and assessment of eligibility of patients with respect to inclusion and exclusion criteria will be done at the recruiting centers.

Inclusion criteria are:

1. Signed and dated informed consent
2. Men and women of any ethnic origin aged 18 years or older
3. Patients with acute STEMI as defined by the universal definition of AMI
4. Successful acute reperfusion therapy (residual stenosis visually <50 % and thrombolysis in myocardial infarction (TIMI) flow ≥2) within 24 hours of symptom onset or thrombolysis within 12 hours of symptom onset followed by successful PCI within 24 hours after thrombolysis
5. LVEF ≤45 % with significant regional wall motion abnormality assessed by quantitative echocardiography 3 to 6 days after reperfusion therapy

Exclusion criteria are:

1. Participation in another clinical trial within 30 days prior to randomization
2. Pregnant or nursing women or women in childbearing age not able to exclude the possibility of a pregnancy
3. Mental condition rendering the patient unable to understand the nature, scope and possible consequences of the study or to follow the protocol
4. Necessity to revascularize additional vessels, outside the target coronary artery, after investigational therapy/placebo administration (additional revascularizations after primary PCI and before investigational therapy/placebo administration are allowed)
5. Persistent cardiogenic shock
6. Known hematological and neoplastic diseases
7. Severe impaired renal function, i.e., eGFR <30 ml/min.
8. Persistent fever or diarrhea not responsive to treatment within 4 weeks prior to screening or severe infection
9. Uncontrolled hypertension (systolic >180 mmHg and diastolic >120 mmHg).
10. Life expectancy of less than 2 years from any non-cardiac cause or neoplastic disease

After ensuring that a patient meets all other eligibility criteria, the investigators will perform the echocardiography, an electronic record of which will be kept and transferred to the central Echocardiography Core Laboratory following analysis. After local quantification of LVEF, randomization will be allowed for all patients with LVEF ≤45 %. As soon as possible after the enrollment the first cardiac magnetic resonance will be performed. At randomization, every patient will receive a number that will be used as the key identifier for the entire study period.

Patients will be randomized to investigational therapy or placebo according to a 1:1 ratio:

- Group 1: placebo on top of optimal standard of care (LVEF will be evaluated by echocontrastography with the infusion of sulfur hexafluoride: SonoVue®, Bracco Milan, Italy) (see protocol in Additional file 1)
- Group 2: administration of subcutaneous Lenograstim (rhu G-CSF, Myelostim 34, Italfarmaco, Milan, Italy) 5 to 10 days after successful reperfusion of the culprit vessel of a large AMI, on top of optimal standard of care (LVEF will be evaluated by echocontrastography with the infusion of sulfur hexafluoride: SonoVue®, Bracco, Milan, Italy) (see protocol in Additional file 1)

Patients with a LVEF >45 % will not be randomized, but will be classified as screening failures. Patients randomized but not treated will be replaced. Patients who discontinue the study after treatment, will not be replaced.

Both in patients treated with Lenograstim and in patients treated with placebo, immediately before the first dose of Lenograstim or placebo and in the morning of the last day of treatment in which the peak of bone marrow-derived stem cell mobilization is expected, an echocardiogram using an echocardiographic contrast agent will be performed. On the days of administration of the drug or placebo, electrocardiogram (ECG), blood pressure, blood count and coagulation analyses will be monitored (see Additional file 1).

In cases of muscle and bone pain or severe headache, patients will be treated with paracetamol.

In the case of leukocytosis (>40,000/mcl) the dose of G-CSF in 24 hours will be halved, and in the case of leukocytosis (>70,000/mcl) completely stopped.

After hospital discharge, all study patients will return to the clinical center for a follow-up visit after 30 days. Six months after randomization all the patients will undergo outpatient cardiac magnetic resonance and conventional echocardiography. At 1 year all patients will attend a final site visit at the clinical centers collecting any clinical event. However, all clinical endpoints will be reported as occurring throughout the follow-up. Major adverse cardio-cerebrovascular events (death, myocardial infarction, stroke and re-hospitalization due to heart failure) at 1 year will be adjudicated by a clinical event committee blinded to the patient treatment allocation. This will ensure a consistent and unbiased adjudication of events across all investigational sites.

### Cardiac magnetic resonance

In the patients enrolled in the study, two different contrast-cardiac magnetic resonance examinations will be performed: the first as soon as possible after the enrollment, the second 6 months after the randomization. Moreover, the patients with severe renal disease will be excluded (glomerular filtration rate <30 ml/min). Cardiac magnetic resonance will be performed on a 1.5-T clinical scanner (1.5 Tesla Magnetom Avanto, Siemens Medical Solutions, Erlangen, Germany) using a phased-array cardiac receiver coil. Additionally, ECG-gated breath-hold cine imaging will be performed.

The cardiac magnetic resonance protocol includes:

1. Morphological and cine evaluation: to determine left ventricular function, using a segmented steady-state free-precession pulse sequence (TrueFISP) in multiple short-axis views every 8 mm by encompassing the left ventricle from base to apex. Typical in-plane resolution is 1.7 × 1.2 × 8 mm; slice thickness: 6 mm; gap: 2 mm. Long-axis view, comparable with standard echo images (two-chamber view, three-chamber view and four-chamber view) will be acquired
2. Myocardial edema evaluation: presence and location of myocardial edema, including intramyocardial hemorrhage will be evaluated on T2-weighted images short-TI inversion-recovery fast spin echo pulse, on consecutive short-axis views. The typical parameters are reported: FOV: 380 to 400 mm; TR: 2 R-R intervals; TE: 100 ms; TI: 150 ms; matrix: 256 × 192; slice thickness: 8 mm
3. Perfusion at rest evaluation: rest first-pass myocardial perfusion will be performed during administration of a gadolinium-based contrast agent (Multihance, 0.2 mmol/kg, Bracco, Milan, Italy) using single-shot saturation recovery gradient-echo pulse sequence, turbo-FLASH sequences. Three short-axis slices will be obtained per heartbeat, every 10 mm, covering the infarct area as seen during cine imaging (TR: 2.1 ms; TE: 1.1 ms; flip angle 12°; time resolution: 1 s; voxel: 2.8 × 2.4 × 10 mm)
4. Early enhancement evaluation: after gadolinium injection, the presence of microvascular damage will be evaluated by T1-weighted 3D inversion recovery gradient-echo sequences. The typical parameters are reported: TR: 4.5 ms; TE: 1.3 ms; flip angle: 158°; slice thickness: 8 mm; gap: 2 mm; matrix: 128 × 256; field of view: 350 mm; pixel size: 1.4 mm/1.4 mm. The images will be acquired on the same slice position used for cine images. The presence of an “early enhancement” will be performed after 2–5 minutes after contrast injection
5. Delayed enhancement evaluation: the presence of myocardial necrosis detected by delayed contrast deposition (“late gadolinium enhancement”) will be evaluated with the same sequences on point 4, but acquired 10–15 minutes after contrast injection. The inversion time will be individually adapted to suppress the remote myocardium signal (typical range from 200 to 300 ms)

After acquisition protocol, the images will be analyzed off-line using a dedicated workstation with a dedicated post-processing software (Circle cvi42). The following parameters will be obtained for each of two CMR acquisitions:

- Analysis of steady-state free-precision sequences: on all short-axis cine slices, the endocardial and epicardial borders will be outlined manually on end-diastolic and end-systolic images. LVEF, end-diastolic and end-systolic volumes, and left ventricular mass will be calculated from the short-axis views
- Analysis of T2-weighted sequences: on T2-weighted images, infarct-related edema was considered present when the signal intensity of the myocardium was >2 standard deviations of the mean signal intensity of the controlateral remote region. The total amount of myocardial edema, representing area at risk, will be expressed as grams and as percentage related to total myocardial mass
- Evaluation of Turbo-FLASH sequences: perfusion defects on “first pass” will be evaluated for each segment according with the 17-segments model
- Delayed enhancement analysis: on delayed enhancement imaging, myocardial infarction will be considered present if the signal intensity of hyperenhanced myocardium is >5 standard deviations of the mean signal intensity of the remote region, and microvascular damage will be defined as a hypoenhanced region within infarcted myocardium. The total amount of myocardial necrosis will be expressed as grams and also as percentage related to total myocardial mass. Moreover, a score will be used to define the extent of necrosis for each segment according with 17-segments model: 1 = necrosis between 0 and 25 % of the entire segment; 2 = necrosis between 25 % and 50 % of the entire segment; 3 = necrosis between 50 % and 75 % of the entire segment; 4 = transmural necrosis extending > 75 % of the entire segment. Finally the amount of salvaged myocardium, expressed as the difference between area at risk on T2-weighted images and extent of delayed enhancement, will be defined.

### Echocardiography

The echocardiographic evaluation will be performed in accordance with the recommendations of the American Society of Echocardiography. The following views should be evaluated during each echo evaluation: parasternal short-axis view, parasternal long-axis view, apical four-chamber view, apical two-chamber view, apical three-chamber view. Quantitative analyses include measurements of left ventricular dimensions: left ventricle end-diastolic/end systolic diameter, LVEDV, LVESD and LVEF, obtained according to Simpson’s rule.

### Myocardial contrast echocardiography

Myocardial contrast echocardiography will be performed in both groups immediately before the first dose of Lenograstim or placebo and on the morning of the last day of treatment. Myocardial contrast echocardiography will be carried out by using a new generation echo machine (iE33, Philips, Milan, Italy), equipped with a 3.5-MHz ultrasound probe and contrast pulse sequencing, which is a software for acquisition and analysis of contrast signal. Analysis of contrast signal relies on a continuous emission of ultrasound and a contemporary comparison between fundamental harmonics deriving from tissues and second harmonics reflected by microbubbles. When using this kind of imaging modality, the image will normally be totally dark prior to contrast administration, as the signal coming from the tissues is nullified and suppressed, whereas the microbubbles’ signal is amplified. A second generation ultrasound contrast agent SonoVue® (Bracco, Milan, Italy) will be administered, according to the market authorization, by peripheral venous access and continuously injected by a dedicated infusion pump (VueJECT, Bracco, Milan, Italy). Continuous infusion provides a constant bubble signal that is fundamental to assess myocardial perfusion. After the destruction of microbubbles by a high mechanical index pulse, myocardial perfusion will be visually assessed during their replenishment of the coronary circulation Images will be acquired as follows: real-time and triggered apical four-chamber view, real-time and triggered apical two-chamber view, real-time and triggered apical three-chamber view, real-time and triggered parasternal short-axis view.

### Duration of the study

Each enrolled patient will remain in the study throughout the entire study duration, with a minimum follow-up of 1 year for each patient. A study patient’s participation may be terminated early for reasonable cause, such as the investigator’s medical decision. At any time the patient has the right to withdraw consent without a negative impact on their medical treatment. However, the investigators are encouraged to ask for the patient’s permission for further follow-up telephone contacts from those whose decision for discontinuation was based on the need for further outpatient visits. Patients will be enrolled during a recruitment phase of approximately 2 years. Since minimum study duration for one patient is 1 year, the overall study duration is approximately 3 years. The Data Monitoring Committee may terminate the study earlier based on safety concerns at any time, or based on the interim efficacy analysis. Competent authorities/ethics committees retain the right of premature termination of the study according to applicable regulations. At an individual study center, the study may be terminated early if the work performed is not compliant with Good Clinical Practice guidelines.

### Statistical methods

The sample size was calculated on the basis of our previous experience, assuming a difference in LVEF of 5 % between the group treated with G-CSF and controls and a standard deviation of 7 %. In addition, considering the novelty of the study one interim analysis will be performed after the enrollment of 60 patients to check presence of a favorable effect of G-CSF + MCE on left ventricular function in comparison to placebo + MCE. Early termination will be possible at this stage if the experimental group show no increase in LVEF fraction in comparison to the control group. However, all patients recruited must complete a 1-year follow-up, even if the trial is stopped prematurely.

All collected data will be summarized by treatment group. Continuous data will be reported by the number of available data points, mean, standard deviation, median and interquartile range. Categorical and ordinal data will be summarized by observed frequencies and percentages relative to the number of non-missing data. For continuous data, comparisons between treatment groups will be done using a two-sample *t* test, or a Wilcoxon rank-sum test if data show serious deviations from a normal distribution. Categorical data will be compared using a chi-square test or Fisher’s exact test, as appropriate. Ordinal data will be compared using a chi-square test for trend.

The primary endpoint of all-cause mortality will be the change in LVEF evaluated by cardiac magnetic resonance. Statistical significance will be claimed when the resulting *p* value is lower than 0.05.

Adverse events, related adverse events and severe adverse events will be summarized by body system and preferred term for the two treatment groups.

## Discussion

In the last two decades, several clinical trials have investigated the effects of G-CSF therapy on cardiac repair: the results collected so far, however, indicate that G-CSF therapy is safe and may be associated with beneficial effects in patients with large AMI. However, the limited amount of evidence is inadequate to reach any definitive conclusions regarding the efficacy of G-CSF therapy.

Therefore, larger randomized clinical trials, with appropriate power calculations, are needed in order to address the current uncertainties regarding this experimental therapeutic approach.

The RIGENERA 2.0 trial, presented here, will test the combined efficacy of G-CSF administration and MCE in improving the regional and global left ventricular contractile function and reducing infarct extension in patients with large AMI, through the homing enhancement of the bone marrow-derived cells within the myocardium. The results of the present study are expected to envision the routine clinical use of this safe, affordable and reproducible approach in AMI patients with left ventricular dysfunction after successful revascularization.

### Trial status

The RIGENERA 2.0 trial received ethical committee approval (Comitato Etico del Policlinico Gemelli, Roma) and AIFA (Agenzia Italiana del Farmaco) approval in July 2015 and has not yet begun patient recruitment.

## Additional file

Additional file 1:
**Procedure schedule and description of study assessments.** Description of data: in this file a detailed description of the study assessments and their schedule is provided. (PDF 224 kb)

## Abbreviations

AMI: acute myocardial infarction
G-CSF: granulocyte-colony stimulating factor
LVEDV: left ventricular end-diastolic volume
LVEF: left ventricular ejection fraction
LVESV: left ventricular end-systolic volume
MCE: myocardial contrast echocardiography
PCI: percutaneous coronary intervention
PROBE: placebo-controlled, randomized, open-label, with blinded evaluation of endpoints
STEMI: ST-elevation myocardial infarction
