# Supplementary material for: The combined effect of subcutaneous granulocyte- colony stimulating factor and myocardial contrast echocardiography with intravenous infusion of sulfur hexafluoride on post-infarction left ventricular function, the RIGENERA 2.0 trial: study protocol for a randomized controlled trial
Source: Trials. 2016 Feb 19;17:97. doi: 10.1186/s13063-016-1172-0 (PMC4759781; doi:10.1186/s13063-016-1172-0)
Supplement: Additional file 1: — Procedure schedule and description of study assessments. Description of data: in this file a detailed description of the study assessments and their schedule is provided. (PDF 224 kb) [file 13063_2016_1172_MOESM1_ESM.pdf]

ADDITIONAL FILE 1

Procedure schedule

Table 1: Schedule of study assessments.

	Screening<br>Days -6 to - 3	Randomization	Treatment<br>Day 0 to 5	Recovery<br>Day 5 to 10	1 month	4-6 Months
Informed Consent	X					
Demographics	X					
Medical History	X					
Killip Class	X				X	X
NYHA Class	X				X	X
Physical Examination	X				X	X
Vital Signs (BP and HR)	X				X	X
ECG	X				X	X
Safety Laboratory	X		X (daily)	X	X	X
Concomitant Medications	X				X	X
Adverse Event / Endpoint Collection			X	X	X	X
Echocardiography+		X				X
Magnetic Resonance		X				X
Recommended but optional Bloods (see below)	X				X	X
Urine Pregnancy test(where applicable)	X				X	X
Investigational therapy			X			X

♦Recommended but optional Bloods Screening:

Hematology: Red blood count, white blood count

Biochemistry and enzymes: serum urea, serum uric acid, glutamic-oxaloacetic transaminase (GOT), glutamic-pyruvate transaminase (GPT), lactate dehydrogenase (LDH), total cholesterol, low density lipoprotein cholesterol (LDL), high density lipoprotein cholesterol (HDL), triglycerides, glycosylated hemoglobin (HbA1c)

Electrolytes: sodium, potassium

Cardiac marker: Creatine kinase (CK), Creatine kinase muscle-brain (CK-MB), (high sensitive) troponin T or I

Coagulation: International Normalised Ratio (INR)

Hematology: hemoglobin, hematocrit, red blood count, white blood count, platelets

Biochemistry and enzymes: serum urea, serum uric acid, GOT, GPT, LDH, total cholesterol, LDL-cholesterol., HDL-cholesterol, triglycerides, HbA1C

Electrolytes: sodium, potassium

Cardiac marker: CK, CK-MB, (high sensitive) troponin T or I

Coagulation: INR

Description of study assessments

Medical history / demographics

A complete medical history will be obtained from each patient at the screening visit. Demographics, including gender, age and ethnic origin, and the smoking status will be recorded.

Killip Class

Patients will be ranked according to the Killip Class status. Definitions of the grading system are as follows:

Class I: Absence of rales over the lung fields and absence of S3. No heart failure. No clinical signs of decompensation.

Class II: Rales over 50% or less of the lung fields or the presence of an S3. *Heart failure*. Diagnostic criteria include rales, S3 gallop and venous hypertension.

Class III: Rales over more than 50% of the lung fields. *Severe heart failure*. Frank pulmonary edema.

Class IV: Cardiogenic shock. Hypotension (a systolic blood pressure of less than 90 mmHg for at least 30 minutes or the need for supportive measures to maintain a systolic blood pressure of greater than or equal to 90 mmHg), end-organ hypoperfusion (cool extremities or a urine output of less than 30 ml/h, and a heart rate of greater than or equal to 60 beats per minute). The hemodynamic criteria are a cardiac index of no more than 2.2 l/min per square meter of body-surface area and a pulmonary capillary wedge pressure of at least 15 mmHg. Signs include hypotension (systolic pressure < 90 mm Hg) and evidence of peripheral vasoconstriction such as oliguria, cyanosis and diaphoresis. Heart failure, often with pulmonary oedema, has also been present in the majority of these patients.

Physical examination

All patients will undergo standard or abbreviated physical examinations. The standard examination is based on the following body systems: body temperature, HEENT (head, eyes, ears, nose, throat), respiratory system, cardiovascular system, abdomen, extremities, neurological system. The abbreviated physical examination consists of body temperature, respiratory system, cardiovascular

system, and lymph nodes. The body weight will be determined at each physical examination. The body height will be obtained at screening only.

Vital signs

Blood pressure and heart rate at rest will be determined using a standard method.

Electrocardiography

A 12 lead electrocardiogram (ECG) will be recorded. ECGs will be recorded while the patient is resting in supine position. Abnormal findings in ECG recordings will be documented and the clinical relevance will be judged. A de-identified copy of the baseline ECG will be digitally stored.

Clinical laboratory

Blood sampling and sample analysis will be performed at the clinical centre. The local investigator is responsible for proper judgment of abnormal blood test results, and is responsible for appropriate patient care following clinically significant pathological results. Please refer to section 6.4.5 to determine whether an abnormal laboratory value may constitute an adverse event.

The following analysis will be obtained at screening, daily during the five days of treatment (from day 0 to day 5) and at 5, 30 and 180 days:

Screening:

Hematology: Hemoglobin, hematocrit, Red blood count, white blood count, platelets

Biochemistry: Serum creatinine, serum urea, serum uric acid, glutamic-oxaloacetic transaminase (GOT), glutamic-pyruvate transaminase (GPT), lactate dehydrogenase (LDH), total cholesterol, low density lipoprotein cholesterol (LDL), high density lipoprotein cholesterol (HDL), triglycerides, glycosylated hemoglobin(HbA1c), Creatine kinase (CK), Creatine kinase muscle-brain(CK-MB),

high sensitive troponin T hs-TnT, Vascular Endothelial Growth Factor (VEGF), Insulin Growth Factor 1 (IGF-1), hepatocyte Growth Factor (HGF), Stromal Derived Factor 1 alpha (SDF-1 alpha)

Urine: Pregnancy test for woman of childbearing potential

Coagulation: Prothrombin Time (PT), International Normalised Ratio (INR), activated partial thromboplastin time (aPTT)

Concomitant medications

All patients participating in the trial should receive treatment according to evidence based guidelines. Concomitant medications will be recorded throughout the study.

Standard Echocardiography

A resting echocardiogram at enrollment and at 4-6 months will be recorded and digitally stored. See details in 5.3.1

Only if the patient qualifies with LVEF ≤ 45% in the local reading, the patients will be randomised

Investigational therapy/placebo

Lenograstim (rhu G-CSF, Myelostim 34, Italfarmaco) (5 µg/kg twice daily for 5 days, subcutaneously) or placebo (saline solution twice daily for 5 days, subcutaneously) will be administered at least 5 days after admission.

Both in patients treated with Lenograstim (rhu G-CSF, Myelostim 34, Italfarmaco) and in patients treated with placebo, immediately before the first dose of Lenograstim (rhu G-CSF, Myelostim 34, Italfarmaco) or placebo and in the morning of the last day of treatment in which the peak of BMSC mobilization is expected, an echocardiogram using an echocardiographic contrast agent (Sonovue, Bracco) will be performed in the days of administration of the drug or placebo, ECG, blood pressure, blood count and coagulation analyses will be monitored.

In the case of muscle and bone pain or severe headache patients will be treated with paracetamol.

In the case of leukocytosis (> 40000/mcl) dose of G-CSF in 24 hours will be halved and in the case of leukocytosis (> 70000/mcl) completely stopped.

Patients will be safely discharged on the basis of laboratory data.
